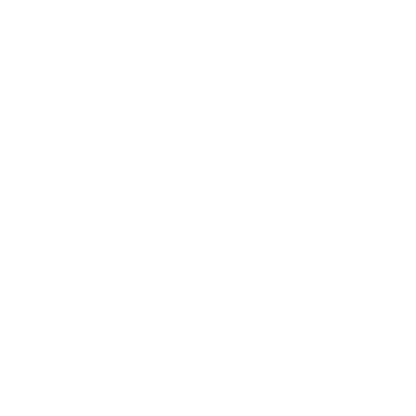# Transparent peer review one year on

**DOI:** 10.1038/ncomms13626

**Published:** 2016-11-10

## Abstract

The majority of our authors are opting in to publish reviewer reports of their papers

Last year we announced a trial of transparent peer review at *Nature Communications* (http://dx.doi.org/10.1038/ncomms10277). In this scheme we publish reviewer reports and author rebuttal letters for papers submitted from January 2016 onwards, but only if authors agree to this upon acceptance of the paper. The anonymity of our reviewers is maintained, unless they sign their reports to the authors. Our intention is to open up the peer review process at the journal, and to provide information on the expert reports that led to our editorial decision to publish a paper. Moreover, the reviewer reports also can serve as a background on the merits of a study, and inform about the discussion between authors and our reviewers. Other steps that we take to support our reviewers are described in a separate editorial (http://dx.doi.org/10.1038/ncomms13625).

In our view, crucial to the success of transparent peer review is the rate at which authors are opting in to the publication of the peer review files. So how did this go so far? The data are shown in Fig. 1 for 787 of the qualifying papers, indexed by their research area.

On average, around 60% of our authors are voluntarily opting in to publishing the peer review history of their paper. We believe that this is a very encouraging result, given also the diversity of our content across so many research areas. Looking at the data for different research fields, not surprisingly, there is some variation. In areas where more open peer review is commonplace and reviewers often sign their reports by name, author opt-in is particularly strong. Other areas may not necessarily be that familiar with publishing reviewer reports, and might therefore still be hesitant to do so. However, in almost all research areas the majority of papers that we publish will now contain a peer review file.

Whereas authors do have the option to opt out of the scheme, this is not the case for reviewers, who have to agree to the potential publication of their reviewer reports. We were curious to see how our reviewers would react to this approach. However, this has only been a problem for a small number of reviewers, who declined to review under this circumstance. For example, we had instances where reviewers objected because they feared that for their small research area it would be easy to deduce their identity from the published reports.

From our readers we have had some feedback, albeit this was more limited as the number of published papers still continues to ramp up. With more time it may also be possible to study the number of downloads of the peer review files in detail. Nevertheless, we have become aware of instances where authors promoted the very positive published reviewer comments of their paper. At the same time, we also had feedback from readers on the peer review discussion of a paper. These instances showcase the potential of transparent peer review—on the one hand to appreciate the value of published papers, and on the other hand to initiate a constructive dialogue between authors and readers on the specifics of a study.

Overall, we are very encouraged by the response to the trial, and will make transparent peer review a permanent feature. We hope that with time more and more authors and readers will appreciate the benefits of having the peer review files published.

Published online: 10 Nov 2016

## Figures and Tables

**Figure 1:**
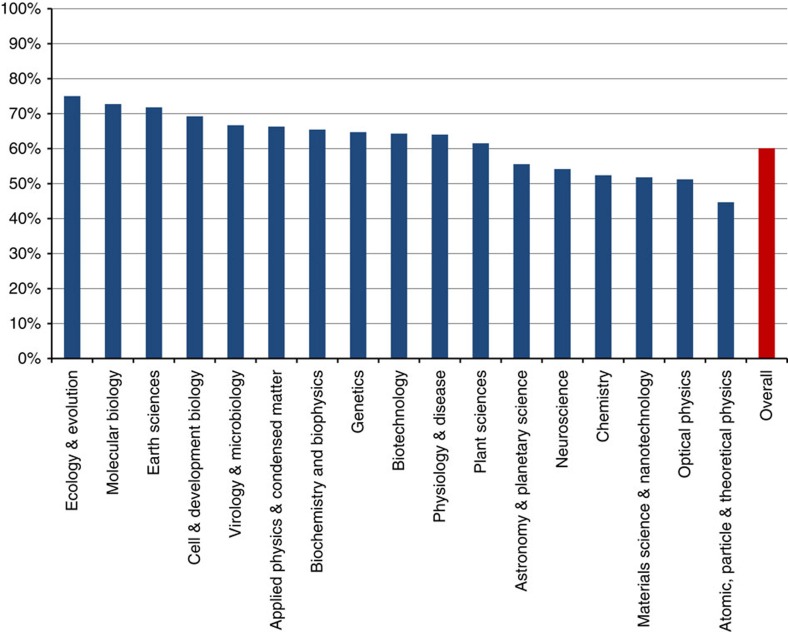
Transparent peer review at Nature Communications. The figure shows the author opt-in rate across the different research areas for 787 published papers. The average opt-in across the journal is about 60%.

**Figure:**